# Epic failure: Lessons learned from interprofessional faculty development

**DOI:** 10.1007/s40037-018-0488-8

**Published:** 2018-11-13

**Authors:** Joy Doll, Anna Maio, Meghan Potthoff

**Affiliations:** Center for Interprofessional Practice, Education and Research (CIPER), Creighton University, Omaha, NE USA

**Keywords:** Interprofessional, Faculty development, Failure

## Abstract

Interprofessional education (IPE) is now recognized as an important initiative to prepare the next generation of health providers. Although IPE has been embraced by many institutions, faculty development still remains an issue. In this manuscript, the authors share their story of one attempt to educate a variety of health science faculty on IPE in what was perceived as an approachable venue. The story of its epic failure and lessons learned will be shared to help others avoid similar pitfalls.

## The story

Since interprofessional education (IPE) and collaborative practice has emerged as a key hot topic and is now infused in the accreditation guidelines for many health professions, faculty development in this area is an evident need. Faculty development for IPE has been identified as critical to the successful implementation of both interprofessional education and practice [1, 2]. In fact, in 2015, the Institute of Medicine released the Interprofessional Learning Continuum which acknowledges a continuum of learning for IPE for learners across the spectrum including health professions education, graduate education and continuing professional development [3]. Without faculty training and recognition of the cultural shift from uniprofessional or multidisciplinary care to interprofessional care, educators continue to perpetuate the gaps and inabilities to reinforce the concepts of IPE.

As an academic institution with significant complexities including eight health professions programs, multiple health sciences schools and colleges and an academic health partner that retains physician employment, the issue of faculty development for IPE seemed almost impossible at our institution. As the IPE leadership team identified the need for a standardized course on topics related to IPE for all early learners across our health sciences programs, the team, a group of faculty members leading IPE at the institution, identified that an online self-paced course promotes the levelling for students prior to engaging in IPE activities. The student course was developed and implemented with success serving almost 1,000 students in its first year. Overall, feedback from the course was positive and it is now entering its third year.

It seemed only natural to create a companion course to this approach for faculty and clinicians. Our team also wanted faculty and clinicians to have a similar interprofessional education to our students. Making them complementary served to ensure our faculty could reinforce and support the student experience. The institution had the benefit of holding an academic Joint Accreditation to provide continuing education across the health professions. With a new academic health partner, it seemed a prime opportunity to create a course both for faculty and clinician learners. In order to promote buy-in and offer a benefit to faculty and clinicians, the team pursed continuing education for the online IPE faculty development course from the Joint Accredited program. Due to the structure and intensity of the course, it was identified as offering 8 continuing education credits and could be a fit for healthcare professions across the spectrum.

The team rolled out the course identifying its benefits, similar to those the students identified including 1) the course is self-paced allowing learners to sit down and engage at their own pace; 2) the course provides a general overview of information on how to collaborate on a healthcare team geared towards multiple health professions; and 3) clinicians and faculty would receive 8 continuing education credits at no cost. All of these factors seemed to our team to be motivators for faculty and clinicians to take the course. Prior to the course release, an opportunity for peer review was offered to faculty and clinicians. Overall, around 40 faculty and staff provided feedback on the peer review and adjustments were made to the course based on this feedback. We then developed an informational email and easy sign-up infrastructure followed by an invitation for faculty and clinicians to take the course.

The team distributed the course to faculty and clinicians hoping to mirror the success of the student course. In addition to offering the course to faculty, the team encouraged the Deans and leadership of the schools and colleges to send out the course with a due date for completion. The course was distributed and the team had high hopes, proud of the product that had been produced and believing it would help faculty become more proficient in IPE.

## Surprising outcomes

Unfortunately, the team’s perceptions of our educational tool were completely wrong. In our minds, we had developed an educational tool that filled a gap for faculty and clinicians where they could earn credits and be better prepared for teaching students. The course’s self-paced nature and ease of access in the institution’s learning management system all seemed like benefits to our team.

Yet, on the course roll out, participation was minimal and dismal. First, many faculty balked at the idea of even needing to learn about IPE and the notion of them benefitting from education on the topic. Complaints rolled in about the enrolment process, course’s length, online format and content, despite being offered for free to all health professions faculty and clinicians in the health system. In the student course, complaints of this nature were minimal, if nonexistent.

Even the opportunity to earn 8 continuing education credits was not an enabler. The only health profession that participated with any depth was nursing because their Dean saw the inherent value and required course participation by a deadline. This same Dean also confirmed faculty participation and sent reminders for course completion. Our team observed a cultural shift after the Nursing faculty completed the course with more buy-in for the IPE student course and multiple faculty reaching out for resources to develop IPE activities. Although other Deans encouraged participation, it was minimal from other health professions. In addition, faculty members and clinicians treated the course creators as the ‘IPE police’ always making excuses to team members as to why they had not participated and freely complaining about the course. Often our team found this aspect surprising as our perspective was to create a resource to help faculty and clinicians be more informed on IPE and the intent was not to hold them to some sort of formal accountability.

Overall, our team made a general assumption that what students perceived as valuable, faculty and clinicians would also value. Our perceptions that developing something accessible and with continuing education credits would be well received was incorrect. In our minds, we were developing an educational resource that would allow flexibility and help faculty support their own professional development. We were truly unaware that faculty, themselves, had spent little time in the learning management system as learners. We did not realize that many viewed IPE as frivolous or the latest healthcare fad and did not feel they needed professional development in this area. After our efforts to ensure an accessible course with credit for faculty and clinicians, we truly were shocked by the negative response and feedback we received across the health professions.

An unintended consequence of putting the course on our IPE website was that more external stakeholders interested in IPE participated in the course. The external stakeholders were individuals at other institutions searching for IPE faculty development and finding our course through an internet search. The positive feedback from external stakeholders further confirmed our beliefs that the course had value and we turned our attention to these individuals to generate revenue for IPE. Although an unintended consequence, this outcome reassured the team that all the hard work and the efforts to assign continuing education was worth it.

## Lessons learned

Overall, upon further reflection, our team has identified four core lessons learned from this experience: 1) an educational success for one group can be a failure in another group; 2) increased cooperation occurs when accountability is built in or the faculty development is made a requirement; 3) champions are critical along with early adopters in leadership; and 4) what your team values as important is not necessarily what others value. Elaborating on each of these, we provide our reflections on this failed experiment on IPE faculty development.

Due to the experience and feedback from students in their version of the course, the team assumed that faculty and clinicians would align with the same benefits. Although we are very aware that students have different accountability measures than faculty and clinicians, our team thought that faculty and clinicians would appreciate the content and delivery method in a similar way to the students. In the student course evaluation, students reported constructively on the ease of the course and the benefits of its self-paced nature. Due to the overwhelmingly positive response from students, our team was surprised at the negative response from faculty and clinicians in the course delivery model. Our failure here was the simple assumption that what would benefit students would also be perceived as beneficial by faculty and clinicians. It demonstrated our lack of recognition of the culture of the faculty buy-in for IPE. We learned that we needed to expand our concept of learners along with considering a unique delivery catered towards each audience rather than a blanketed approach.

Our team discovered that faculty and clinicians did not have an inherent desire to gain knowledge in IPE. Without a clear tie to their current faculty or clinical role or a clear incentive or requirement, faculty and clinicians were not going to complete the course. Besides nursing, where the course is required, those from other professions who completed the course found value and recognized a need to learn about IPE. Essentially, mostly early adopters engaged in the course, those already recognizing a value for IPE. Overall, IPE has struggled with its perceived distance and impact on clinical practice [4]. The lack of a clear tie to clinical practice most likely impacted clinician enrolment in our course. Our team was repeatedly told busy clinicians do not have time for such training. Although the developers cannot identify this as the critical factor for lack of participation, the perceived lack of value of IPE across healthcare professionals still exists and permeated our experience. However, we know a gap exists for clinicians who are not graduating ‘collaboration ready’ for a collaborative care environment [5]. In fact, most training of clinicians has focused on select teams and not large groups including the work of Team STEPPS [6]. Essentially, we learned that a large scale IPE faculty development initiative will face challenges if the culture of the institution is not ready or supportive. Additionally, we might have improved the overall participation if we had taken more time to assess the learning needs of our faculty and clinicians. We developed the course based on our perceptions of the knowledge needed to engage in successful IPE. Despite offering a peer review and developing the course with an interprofessional team, we could have further assessed the gaps in knowledge to design something clinicians and faculty really needed.

To get the course developed and implemented, champions are critical. The need for champions for IPE has been clearly documented as critical to success [7, 8]. The Dean of the College of Nursing is a clear example. Only our nursing faculty completed the course in mass and completion was due to accountability along with a clear deadline by the Dean of that health profession. Participation from nursing is still ongoing for new nursing faculty. Essentially, the course became a requirement of faculty development and onboarding in the College of Nursing. This leader’s willingness to make the course required ensured participation and overall, the team received less complaints from these stakeholders than others. Without leadership, participation among nursing would likely have been similar to the other health professions. Champions and administrative support for IPE has been clearly identified as critical to successful IPE faculty development by others in this field [2].

Early adopters are also important. Suter and colleagues [9] identified that using Rogers Diffusion of Innovation Theory can be an asset to implementing IPE. In this model, Rogers acknowledges that when innovating, participants fall into camps including innovators, early adopters, early majority, late majority and laggards [10]. Although at times this theory has been identified as simplified, it resonates with our team [9]. Our team was the innovators and we certainly saw individuals fall into the other categories. We learned that early adopters who engage and then share the value of the experience help engage those in the other categories. As a team, we learned that it is a process and takes time to recognize where everyone lands in their willingness to adapt to change. We became more sensitive to members of our community’s capacity for change. Moving forward, our IPE faculty development initiatives became more catered to the learners in their context and more individualized. We also built in accountability measures identifying that for any approved IPE curriculum activity for students, we must require at least one faculty or clinical member to successfully complete our IPE faculty development course to demonstrate their competence in leading the IPE activity.

Perhaps our biggest mistake was assuming that our passions for IPE were shared by others. Our own ethnocentrism blinded us to the real culture of the institution towards IPE. Each member of our team is admittedly a champion for IPE and immersed in its value. Perhaps we thought we were preaching to the choir and lacked the recognition of the true culture of the institution at the time. In a way interprofessionalism is controversial and we neglected to think about how our faculty development efforts would stir up the many cultural nuances that occur in collaboration among health professions. This concept has been echoed in the literature and our team was naïve to the cultural implications of IPE faculty development due to our closeness and passion to the topic [11, 12]. Through this experience we learned to be much more sensitive and follow a design thinking approach focused on empathy and support as we promote a culture shift towards more interprofessional education and collaboration [4]. We acknowledge our gap in truly assessing our learner needs related to faculty and clinicians.

## Moral of the story

The moral of the story is that leaders in IPE should obtain a pulse of the culture of the institution and ask not just champions and early adopters about the best approaches for IPE faculty development. Proceeding forward with assumptions that the student experience will mirror the faculty development process puts a team at risk for failure. Leaders in IPE faculty development should recognize their institution’s culture and expect both early adopters and laggards.
